# Attention-deficit/hyperactivity disorder and dementia – is there a link?

**DOI:** 10.1192/j.eurpsy.2023.552

**Published:** 2023-07-19

**Authors:** C. Pinheiro Ramos, M. Magalhães, C. Baptista, R. Ribeiro, A. Gamito

**Affiliations:** Psychiatry and Mental Health Department, Setúbal Hospital Center, Setúbal, Portugal

## Abstract

**Introduction:**

Attention-deficit hyperactivity disorder (ADHD) is a neurodevelopmental disorder characterized by cognitive deficits and/or behavioral disturbances. The symptoms begin before 12 years and must cause an impact in different contexts. It is now recognized that in 40–60% of cases, ADHD symptoms persist into adulthood and old age, representing nearly 4% of adults and seniors.

Executive and memory deficits have been described in other neurodevelopmental disorders, such as autism, and older adults with these disorders are observed, later in life, with mild cognitive impairment (MCI) or dementia.

MCI is conceptualized as a prodromal stage of a neurodegenerative process, for which the pathological processes are not yet known. The term “MCI” is currently used to designate subjective complaints and performance below expected levels, in any cognitive domain.

There is, therefore, an overlap between ADHD and MCI in older adults, related to cognitive and behavioral symptoms. This overlap makes both syndromes difficult to distinguish, particularly in older patients.

**Objectives:**

To highlight the importance of understanding the key processes of ADHD and MCI and how these entities may be related to each other.

**Methods:**

Non-systematic review of the literature using *Pubmed* database. Papers were selected according to their relevance.

**Results:**

Sleep disturbances are present in about 70% of adults with ADHD, and 59% of those with MCI. Depression and anxiety, respectively, are observed in about 44% and 35% of adults with ADHD, and 27% and 14% of those with MCI.

In the literature, the relationship between ADHD and MCI/Dementia remains unclear, although there are some hypotheses: (a) ADHD and MCI represent two points along a single pathophysiological continuum; (b) ADHD increases the risk for MCI and dementia (through an unrelated mediator); (c) ADHD and MCI manifest highly similar neurobehavioral symptoms through fundamentally distinct mechanisms (are unrelated). However, these three hypotheses are not mutually exclusive, i.e. ADHD may share common antecedent causal factors with MCI/Dementia and also increase the risk of MCI/Dementia through an unrelated mediator.

Neuroimaging evidence tends to support the hypothesis that neurobehavioral symptoms in ADHD and MCI manifest via distinct processes within the brain, with frontostriatal, frontal-temporo-parietal, and fronto-cerebellar abnormal networks in ADHD and progressive neurodegeneration in MCI.

**Conclusions:**

Whether or not ADHD is a phase of a neurodegenerative process, the current criteria for the diagnosis of MCI or Dementia may not be appropriate or valid in individuals with a premorbid history of ADHD.

The criteria for the diagnosis of MCI/Dementia in adults with a previous diagnosis of ADHD should therefore be revised to rely more on functional outcomes.

Future neurobiological and epidemiological studies are needed, to explore the relationship between MCI/Dementia and ADHD, in older adults.

**Disclosure of Interest:**

None Declared

